# Curcumin Prevents Palmitoylation of Integrin β4 in Breast Cancer Cells

**DOI:** 10.1371/journal.pone.0125399

**Published:** 2015-05-04

**Authors:** David T. Coleman, Young Hwa Soung, Young-Joon Surh, James A. Cardelli, Jun Chung

**Affiliations:** 1 Department of Physiology and Stephenson Cancer Center, The University of Oklahoma Health Sciences Center, Oklahoma City, Oklahoma, United States of America; 2 Department of Microbiology and FeistWeiller Cancer Center, Louisiana State University Health Sciences Center, Shreveport, Louisiana, United States of America; 3 College of Pharmacy, Seoul National University, Seoul, South Korea; Winship Cancer Institute of Emory University, UNITED STATES

## Abstract

Curcumin has been shown to mitigate cancer phenotypes such as invasive migration, proliferation, and survival by disrupting numerous signaling pathways. Our previous studies showed that curcumin inhibits integrin β4 (ITG β4)-dependent migration by blocking interaction of this integrin with growth factor receptors in lipid rafts. In the current study, we investigated the possibility that curcumin inhibits ITG β4 palmitoylation, a post-translational modification required for its lipid raft localization and signaling activity. We found that the levels of ITG β4 palmitoylation correlated with the invasive potential of breast cancer cells, and that curcumin effectively reduced the levels of ITG β4 palmitoylation in invasive breast cancer cells. Through studies of ITG β4 palmitoylation kinetics, we concluded curcumin suppressed palmitoylation independent of growth factor-induced phosphorylation of key ITG β4 Ser and Tyr residues. Rather, curcumin blocked autoacylation of the palmitoyl acyltransferase DHHC3 that is responsible for ITG β4 palmitoylation. Moreover, these data reveal that curcumin is able to prevent the palmitoylation of a subset of proteins, but not indiscriminately bind to and block all cysteines from modifications. Our studies reveal a novel paradigm for curcumin to account for much of its biological activity, and specifically, how it is able to suppress the signaling function of ITG β4 in breast cancer cells.

## Introduction

Curcumin [chemical name: 1,7-bis(4-hydroxy-3-methoxyphenyl)-1,6-hepadiene-3,5 dione], is a natural polyphenol component of turmeric (*Curcuma longa*) with demonstrated biological activity against a wide assortment of human pathologies. Chemopreventive and therapeutic properties of curcumin have been well established *in vitro* and *in vivo* for various types of human cancer [1,2]. The ability of curcumin to act selectively on cancer cells over normal cells highlights its potential as a useful cancer preventive or therapeutic modality with minimal toxicity [3]. However, the molecular mechanism by which curcumin influences various, seemingly unrelated, signaling pathways to selectively obstruct aggressive cancer cells is unknown.

In previous reports, we showed that integrin β4 (ITG β4) is a target of curcumin in aggressive breast cancer cells [4]. Curcumin inhibited ITG β4-dependent signaling events important for breast cancer cell motility, invasion and anchorage-independent growth [4]. Our subsequent studies demonstrated that inhibition of ITG β4 signaling by curcumin is mediated by blocking localization of ITG β4 to lipid raft membrane domains and disrupting its interaction with growth factor receptors [5]. Mobilization of ITG β4 from hemidesmosomes into the leading edges and lipid rafts is thought to play an essential role for signaling competency of this integrin [6–8].

Post-translational modifications of the ITG β4 cytoplasmic domain play an important role in its trafficking and signaling competency. For example, a number of reports have shown that phosphorylation of key tyrosine and serine residues along the cytoplasmic tail contributes to hemidesmosome disassembly and subsequent activation of ITG β4 signaling in human cancer cells [9–11]. ITG β4 is also palmitoylated on five cysteine residues (C732/736/738/739/742) along a juxtamembrane segment of the cytoplasmic tail through a reversible thioester linkage to regulate its signaling and trafficking [8, 9–11]. The palmitoylation of ITG β4 is catalyzed by the palmitoyl acyltransferase DHHC3 [12, 13]. Palmitoylation of ITG β4 is necessary for incorporation of α6β4 into lipid rafts, where ITG β4 crosstalks with growth factor receptors and enhances invasive potential of cancer cells [5, 8]. Importantly, an alternative report suggests palmitoylation of ITG β4 is not required for raft association, but rather for incorporation into tetraspanin complexes [14]. In either scenario, the palmitoylation of ITG β4 mediates its trafficking important for signaling competency.

In the present study, we examined the hypothesis that curcumin blocks ITG β4 trafficking and signaling competency by blocking its palmitoylation. We demonstrated that curcumin blocked the palmitoylation of ITG β4 in invasive breast cancer cells exhibiting high basal levels of ITG β4 palmitoylation. Investigating the kinetics of ITG β4 palmitoylation in the context of phosphorylation events, we were able to determine curcumin had a direct effect on palmitoylation. Further experimentation revealed curcumin inhibits palmitoylation of multiple proteins by interfering with the autoacylation intermediate step of DHHC palmitoylating enzyme activity. Overall, this work supports a novel paradigm to explain the seemingly pleiotropic biological activity of curcumin in aggressive cancer cells.

## Experimental Procedures

### Cell lines and reagents

MDA-MB-231 and MDA-MB-435, cells were obtained from the Lombardi Breast Cancer Depository at Georgetown University (Washington, DC). and were cultured in DMEM with 10% fetal bovine serum and 1% antibiotics. Both HCC-1806 breast cancer cells, cultured in RPMI-1640 with 10% fetal bovine serum, and MCF-10A breast epithelial cells, cultured in MEGM containing 13 mg/ml BPE, 0.5mg hydrocortisone, 10 μg/ml hEGF, 5 mg/ml insulin and 100 ng/ml Cholera toxin (Lonza), were purchased from ATCC. All cell lines were maintained in humidified incubators at 37°C in 5% CO_2_.

β4 Integrin (clone H-101), and actin (clone C-11) antibodies were purchased from Santa Cruz Biotechnology (Santa Cruz). GFP monoclonal antibody was obtained from Clontech. Myc-tag (clone 71D10), Phospho-PKC (pan), Phospho-PKC α/β and Phospho-PKC δ were purchased from Cell Signaling. Phospho-β4 integrin (Y1494) from ECM Biosciences. Flotilin-2, Syntaxin-6 antibodies were from BD Transduction. DHHC3 (GODZ) antibody was purchased from Abcam. Tubulin antibody was from Neo markers. Transferrin receptor antibody was from Invitrogen. Anti-GSH antibody, Dithiothreitol (DTT), *N*-ethylmaleimide (NEM), hydroxylamine (HAM), curcumin, and 2-bromopalmitate were from Sigma-Aldrich. EZ-link BMCC-Biotin and Streptavidin-HRP were purchased from Thermo Scientific. Tetrahydrocurcumin (THC) was provided by Dr. Surh (Research Institute of Pharmaceutical Sciences, Seoul National University). For pharmacologic inhibition, Go6976 (PKC-α, -β, -γ isoforms inhibitor), Go6983 (PKC-α, -β, -δ, -γ isoforms inhibitor) and BIM-1 (pan PKC inhibitor) were purchased from Enzo Life Sciences. PP2 (Src inhibitor) was obtained from EMD chemicals Inc.

### Plasmid constructions and transfection

Wild-type β4 integrin in the vector pRc/CMV was gift from Dr. Shaw Leslie. cDNA of β4 WT was sub-cloned into the c-terminal GFP tagged pcDNA3 and Myc tagged pCMV-Tag 5 plasmid (Agilent Technologies) using standard techniques. Five cysteine mutations (C/A 732,736,738,739,742), triple serine mutations (S/A 1356,1360,1364) or S/A, S/D 1424 and single serine mutations (Ser 1424 to Ala or Asp) of ITG β4 were made by Mutagenex, Inc (Piscataway, NJ). The constructs were digested by the EcoRI/SalI restriction sites and then sub-cloned into the c-terminal GFP tagged pcDNA3 or Myc tagged pCMV- Tag 5. The expression plasmid encoding HA tagged DHHC3 was purchased from the GeneCopoeia, Inc. All plasmids were verified by sequencing (Macrogen USA) and western blotting. Transfection of the plasmids was performed using Lipofectamine LTX-Plus (Invitrogen) according to the manufacturer’s instruction.

### Western analysis

Cells were lysed in cold RIPA-EDTA buffer [50 mMTris, pH 7.4; 150 mMNaCl; 1% NP-40; 0.5% sodium deoxycholate; 0.1% SDS; and 5 mM EDTA] containing 1 mM PMSF, 1 mM Na_3_VO_4_, and protease inhibitor cocktail (Thermo Scientific, Rockford, IL). Protein concentrations were determined using the BCA protein assay kit (Thermo Scientific). Samples diluted in Laemmli running buffer with BME were separated by SDS/PAGE and transferred to PVDF membrane (Bio-Rad, Hercules, CA). Membranes were incubated with primary antibodies in TBS-T with 5% nonfat dry milk overnight at 4°C, washed and then incubated with appropriate HRP-conjugated secondary antibodies. Proteins were detected using the pierce ECL western blotting substrate (Thermo Scientific).

### Invasion assay

Invasion assays were performed by a transwell cell culture chamber of 8 μm pore size (BD Falcon, Franklin Lakes, NJ) according to the standard procedure. Transwell inserts (8 μm pore size) were coated with collagen I (15 μg/ml) overnight at 4°C. After washing with PBS, indicated cells (1x10^5^cells/well) were treated with or without 15 μM curcumin added to the insert in serum-free DMEM/BSA. Lysophosphatidic acid (LPA, 100 nM) was added to the lower chamber in serum-free DMEM/BSA as a chemo-attractant. The chamber was incubated for 2.5 hours under cell culture conditions. Migrated cells attached to the underside of the membrane were stained with crystal violet and counted. Assays were performed in triplicate and repeated three times.

### Acyl-Biotin Exchange

The acyl-biotin exchange technique was performed as previously described [15]. In brief, cells were treated with or without curcumin for 5 hours prior to lysis (150mMNaCl, 5mM EDTA, 50mM Tris HCl, .02% NaN_3_, 2% Triton X-100, pH7.4, with Roche protease inhibitor cocktail). Equal protein was aliquoted for each sample and all unmodified free cysteine residues were covalently blocked by 50 mM N-ethylmaliamide treatment at pH 7.2 for 24 hours. Protein was methanol-chloroform precipitated and washed 3 times in methanol. Protein was resuspended in 1% SDS 50mM Tris-HCl pH 7.4 with or without hydroxylamine (HAM) for 1 hour at room temperature rotating end-over-end to remove thioester-linked cysteine modifications. Protein was again precipitated and washed. Now-free cysteines were bound by biotin (1 μM EZ-Link Biotin-BMCC) at pH 7 for 24 hours at 4°C rotating. Protein was precipitated and washed before resuspending in non-reducing Laemmli buffer and boiling for 3 mins. Samples were separated by SDS-PAGE and blotted with streptavidin-HRP to detect the presence of biotinylated (S-acylated) protein.

### Click chemistry-based detection of palmitoylated or nascent protein

Cell lines were grown to 80% confluency in 10 cm dishes. All dishes were pretreated with culture media containing 10% fatty acid free FBS (Gemini) with DMSO or 15 μM curucmin for 1 hour. Media is then replaced with fresh fatty acid free culture media containing alkyne-labeled palmitic acid (17-ODYA, Caymen Chemicals) with or without 15 μM curcumin. Cells were incubated for 4 hours to allow incorporation of labeled fatty acids. Cells were lysed in NP-40 buffer (25mM Tris-HCl, pH 7.4, 150mM NaCl, 1mM EDTA, 1% NP-40 and 5% glycerol with Roche protease inhibitor cocktail tablets) at 4°C. Lysates are processed for 30 minutes with end-over-end rotation at 4°C followed by removal of debris by centrifugation at 13,000xg for 5 minutes. Protein concentrations were determined and 200 μg of each sample was methanol-chloroform precipitated. Precipitated protein was resuspended in 50 μl of 1% SDS reaction buffer and the click chemistry reaction was performed according to the manufacturer's protocol using the Click-iT reaction buffer kit and corresponding biotin-based detection reagent (Invitrogen). Following linkage of labeled fatty acid (17-ODYA) to biotin, protein was again precipitated and resuspended in 100 μl of 1% SDS. Once protein was solubilized, SDS was diluted out with 1 mL of NP-40 buffer. Samples were precleared for 1 hour at 4°C with A/G sepharose beads (Santa Cruz). After preclear, 100 μl of prewashed streptavidin-conjugated sepharose beads were added to each sample to pull down biotinylated protein. Samples were rotated end-over-end overnight at 4°C. Pull-down samples were washed 4 times in IP buffer, before protein was eluted with 30 μl of Laemmli buffer containing BME. Samples were analyzed by western blot using indicated antibodies.

For detection of newly synthesized protein, dishes were incubated in Methionine-free growth media (Invitrogen) containing 10% FBS for 1 hour prior to treatments. Media was then replaced with fresh Methionine-free growth media containing 10% FBS with or without 30 μM azido-homoalanine (AHA) (Invitrogen) in the presence or absence or curcumin to label nascent proteins. Processing of lysates, click chemistry reaction, and pull-down of biotinylated protein was performed as detailed above.

## Results

### Curcumin blocks palmitoylation of ITG β4

**Fig 1:**
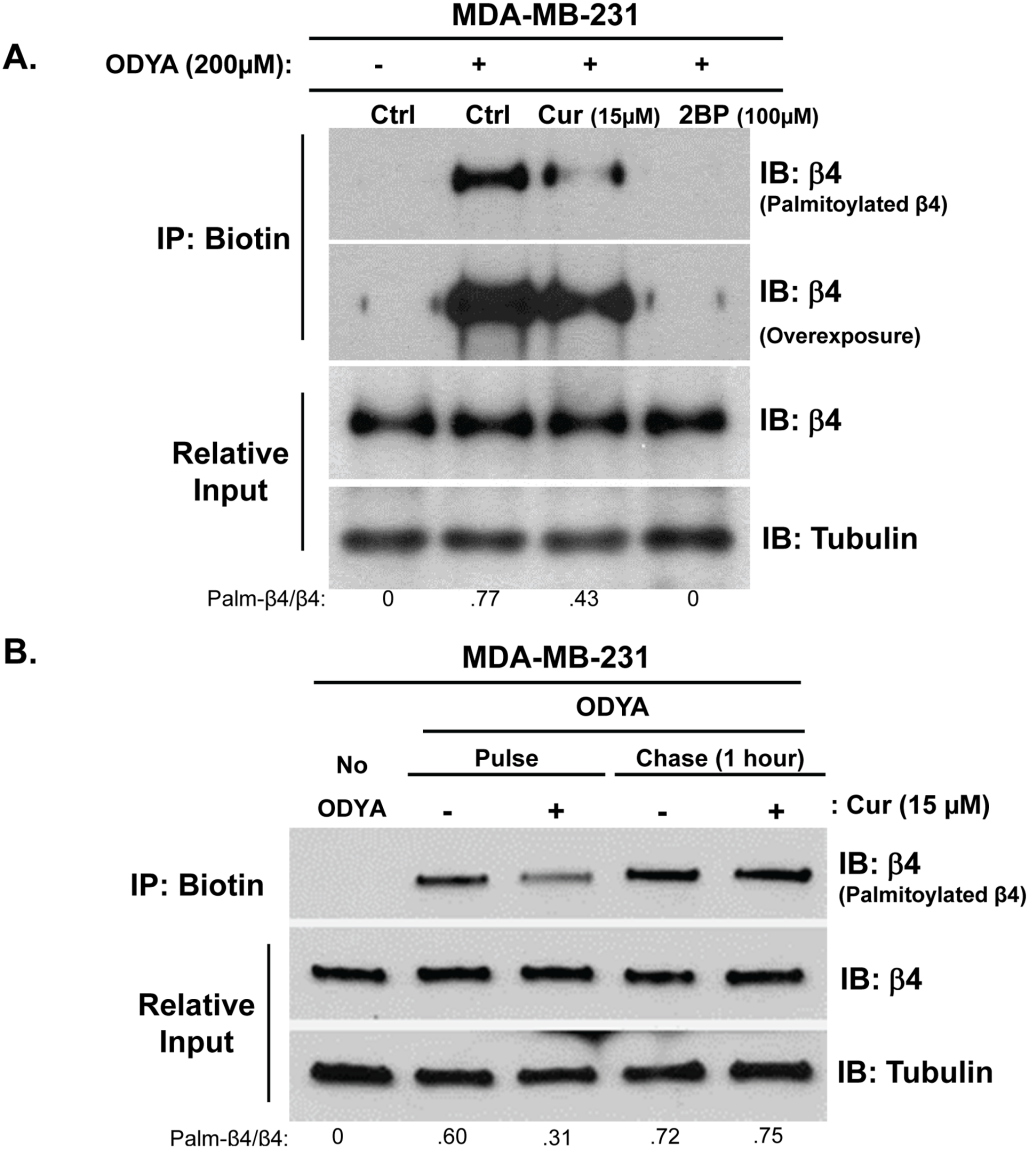
Curcumin inhibits palmitoylation of ITG β4. (A) MDA-MB-231 cells were pretreated with 15 μM curcumin or 100 μM 2-BP (2-bromopalmitate) for 1hr prior to 17-ODYA labeling for 4 hr in the presence of either compound. (B) Cells were either treated with 15 μM curcumin1 hr before and during 17-ODYA labeling for 3 hours, or labeled with 17-ODYA for 3 hours followed by wash and 4 hours chase treatment with 15 μM curcumin. Labeled proteins were reacted to biotin-azide via click chemistry and biotinylated proteins were isolated using streptavidin-sepharose. Palmitoylated ITG β4 was detected by western blot analysis using anti-β4 antibody. Representative blots of 3 independent experiments are displayed with relative input protein included. Densitometry is shown in arbitrary units.

Our previous work showed that inhibition of ITG β4 signaling by curcumin is mediated through blocking its lipid raft localization, thereby reducing its interaction with signaling receptors such as EGFR [4, 5]. However, a detailed molecular mechanism by which curcumin inhibits the mobilization of ITG β4 into the lipid rafts remains to be determined. Based on the previous report that the lipid raft localization of ITG β4 requires palmitoylation of transmembrane proximal cysteine residues [8], we tested whether curcumin affected palmitoylation of ITG β4. MDA-MB-231 cells were metabolically labeling with or without the alkyne-linked palmitate analogue, 17-ODYA [16,17], in the presence or absence of curcumin for 5 hours. Lysates were processed through a click chemistry-based alkyne to biotin conjugation and streptavidin-based pull-down assay to measure protein palmitoylation. As shown in Fig 1A, the baseline palmitoylation level of ITG β4 was detected in MDA-MB-231 breast cancer cells, and curcumin reduced ITG β4 palmitoylation similar to the level observed in cells treated with the palmitoylation inhibitor 2-bromopalmitate (2BP). For each experiment, a no ODYA control is included to indicate the non-specific background intensity following the click chemistry reaction and streptavidin-based pull-down. In order to determine if curcumin was able to reverse palmitoylation or simply block incorporation of labeled palmitate, cells were pulsed and chased with or without 17-ODYA in the presence or absence of curcumin. The results indicated that curcumin was consistently able to block palmitoylation of ITG β4, but was not able to reverse the attachment of labeled palmitate (Fig 1B).

### Blockade of ITG β4 palmitoylation by curcumin is more pronounced in invasive breast cancer cells

**Fig 2:**
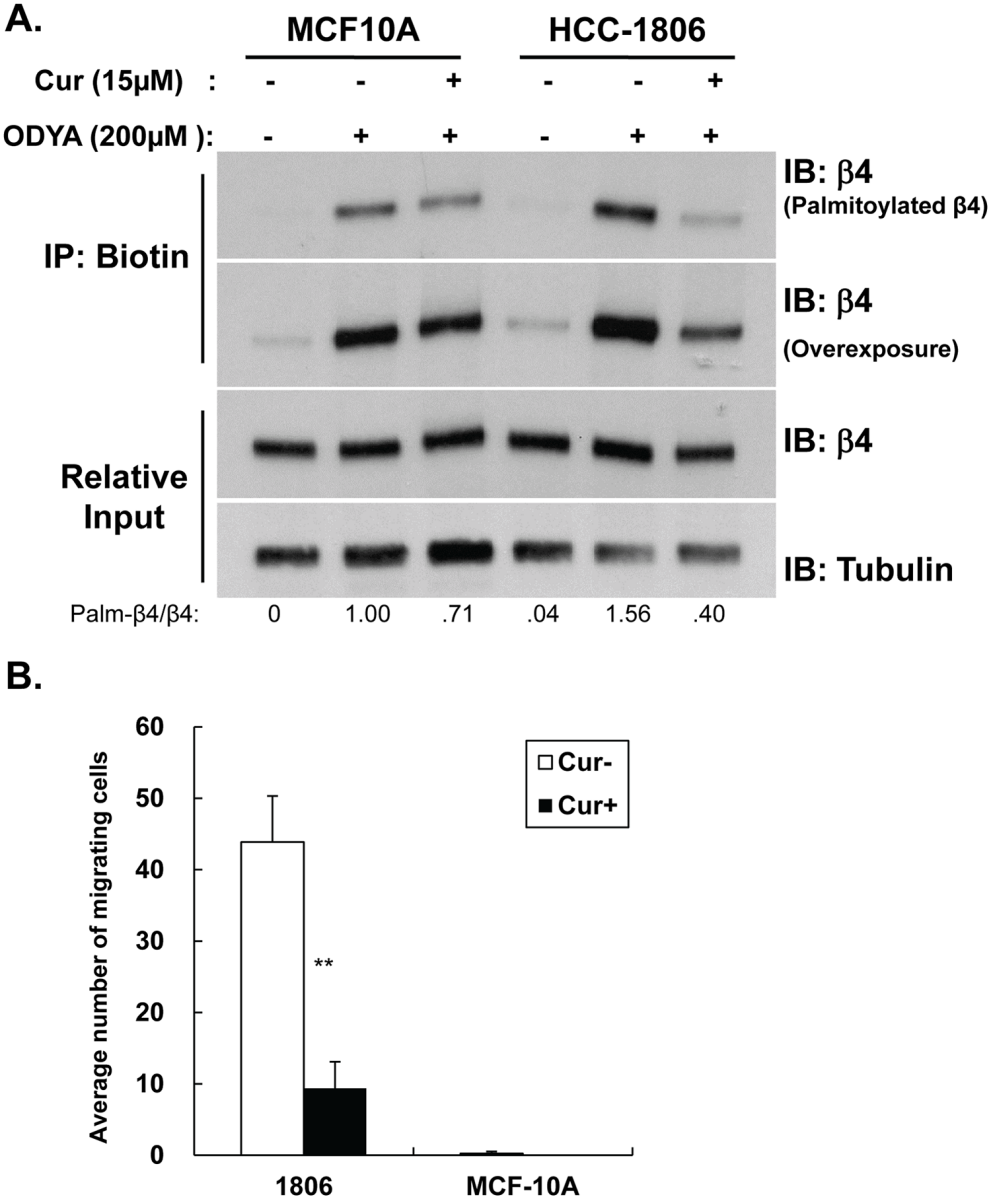
Inhibition of ITG β4 palmitoylation by curcumin is more effective in invasive cancer cells. (A) MCF-10A and HCC-1806 cells pretreated for 1 hour with or without 15 μM curcumin and labeled with 17-ODYA for hours with or without 15 μM curcumin. Labeled proteins were reacted with biotin-azide via click chemistry, isolated by streptavidin-sepharose, and analyzed by western blotting with anti-β4 antibody. Results are representative of 3 independent experiments. Densitometry is shown in arbitrary units. (B) MCF-10A and HCC-1806 cells were incubated with or without 15 μM curcumin. The ability of cells to invade toward 100 nM LPA was measured using a collagen-coated transwell assay. Invasion was quantified by counting cells that migrated to the lower surface of the membrane per square milliliter using bright-field optics. *Columns*, mean from three independent experiments; bars, SD. **, *P < 0*.*01*, versus control groups (without curcumin).

Based on our previous study that curcumin inhibits cell motility and invasion in cancer cells expressing signaling competent ITG β4 [5], we compared the effect of curcumin on ITG β4 palmitoylation in an invasive breast cancer cell line (HCC-1806) and a non-invasive normal epithelial cell line (MCF-10A). These two cell lines express comparable levels of ITG β4, but, based on Y1494 phosphorylation status and previous reports, only the ITG β4 from HCC-1806 is in a signaling competent form under basal conditions (data not shown) [9, 18]. As shown in Fig 2A, less ITG β4 was palmitoylated in the normal epithelial cell line MCF10A as compared to palmitoylation levels observed from HCC-1806 cells. Similar to results from MDA-MB-231 cells, curcumin reduced the level of ITG β4 palmitoylation in HCC-1806 cells, whereas it had little effect on ITG β4 palmitoylation in MCF-10A cells particularly as the basal level of ITG β4 palmitoylation was already low (Fig 2A). Moreover, the ability of these two cell lines to invade towards LPA in transwell assays trended with the palmitoylation levels of ITG β4 (Fig 2B). These data further support a hypothesis that ITG β4 palmitoylation is important for the invasive potential of cancer cells and provides a basis for the previously reported selective activity of curcumin on invasive cancer cells over normal cells.

### The unsaturated aliphatic chain of curcumin is essential for inhibition of ITG β4 signaling and palmitoylation

**Fig 3:**
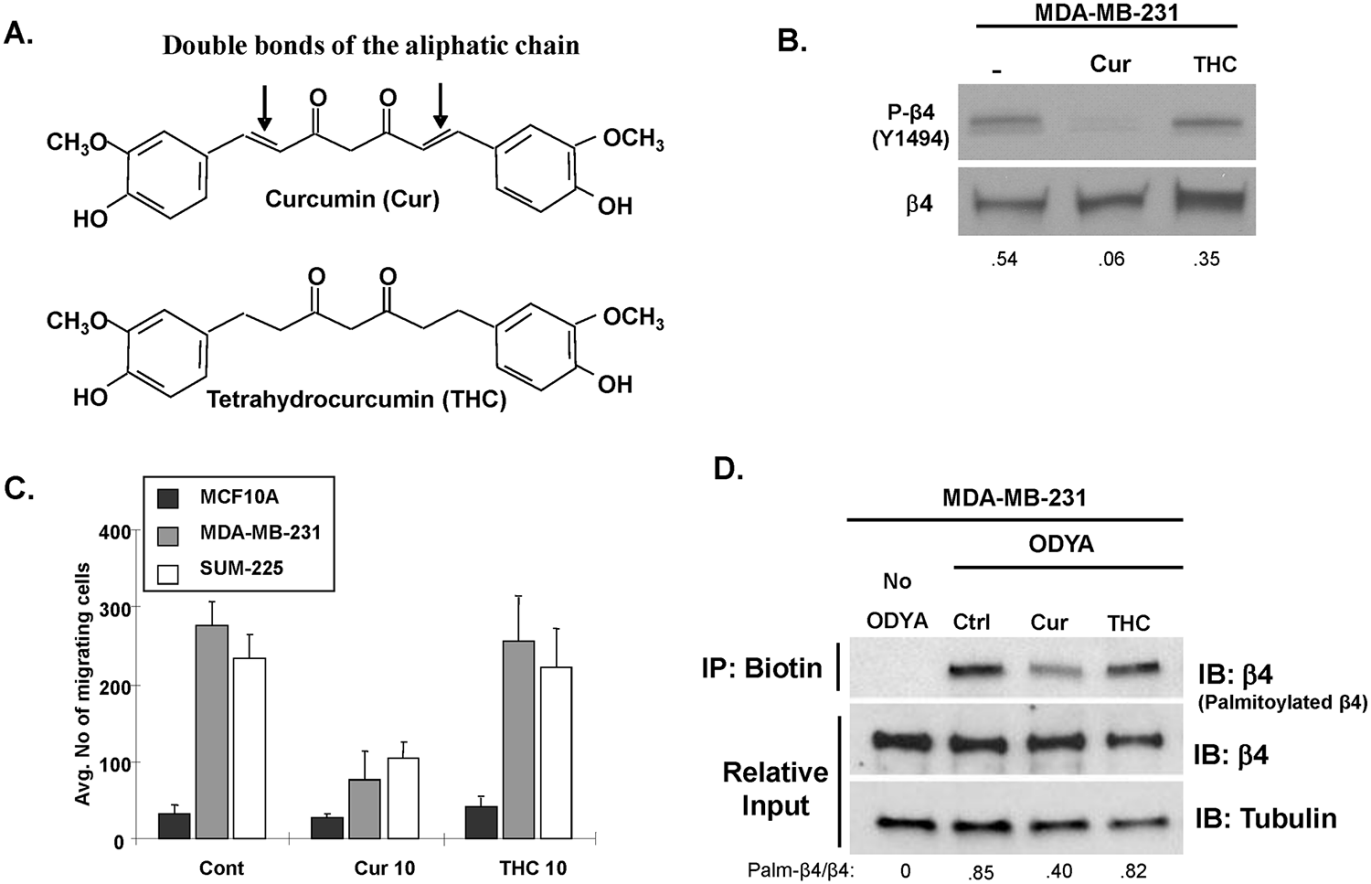
The unsaturated aliphatic chain of curcumin is essential for inhibition of ITG β4 signaling and palmitoylation. (A) Schematic illustrating structural differences between curcumin and THC emphasizing the saturation state of the aliphatic chain. (B) MDA-MB-231 cells incubated with 10 μM curcumin or THC for 24 hours. Lysates were taken and western blot analysis was performed with immunoblotting for the indicated proteins. (C)MCF-10A, MDA-MB-231, and SUM-225 cells were incubated with or without 15 μM curcumin or 15 μM THC. The ability of cells to migrate toward 100 nM LPA was measured using a transwell cell motility assay. Invasion was quantified by counting cells that migrated to the lower surface of the membrane per square milliliter using bright-field optics. *Columns*, mean from three independent experiments; bars, SD. **, *P < 0*.*01*, versus control groups (without curcumin). (D) MDA-MB-231 cells were pretreated with 15 μM curcumin or THC for 1hr prior to or during 4 hr of 17-ODYA labeling. Labeled proteins were reacted to biotin-azide via click chemistry and biotinylated proteins were isolated using streptavidin-sepharose. Palmitoylated ITG β4 was detected by western blot analysis using anti-β4 antibody. Representative blots of 3 independent experiments are displayed with relative input protein included and densitometry shown in arbitrary units.

Curcumin possess two aromatic rings connected by α\β-unsaturated carbonyl moieties [1,19]. These unsaturated double bonds in the aliphatic chains of curcumin have been shown to be critical for its antioxidant and anticancer activity as well as its ability to undergo a Michael addition reaction with accessible cysteine residues [20–22]. To address the role curcumin’s unsaturated aliphatic chain in blocking ITG β4 palmitoylation and signaling competency, we compared the effects of tetrahydro-curcumin (THC), a curcumin analogue with a saturated aliphatic chain, to curcumin [20, 21] (Fig 3A). Unlike curcumin, THC was unable to reduce Y1494 phosphorylation of β4 (an indicator of ITG β4 signaling competency: Fig 3B) in MDA-MB-231 cells or block invasion of the invasive breast cancer lines MDA-MB-231 and SUM-225 (Fig 3C). Consistent with these findings, THC also failed to reduce palmitoylation levels of ITG β4 in MDA-MB-231 cells to the same extent as curcumin (Fig 3D). Taken together, these data indicate that the saturation status of the aliphatic chain in curcumin plays a critical role in obstructing ITG β4 signaling and palmitoylation.

### Integrin β4 palmitoylation does not require growth factor stimulation or phosphorylation of its key Ser or Tyr residues

**Fig 4:**
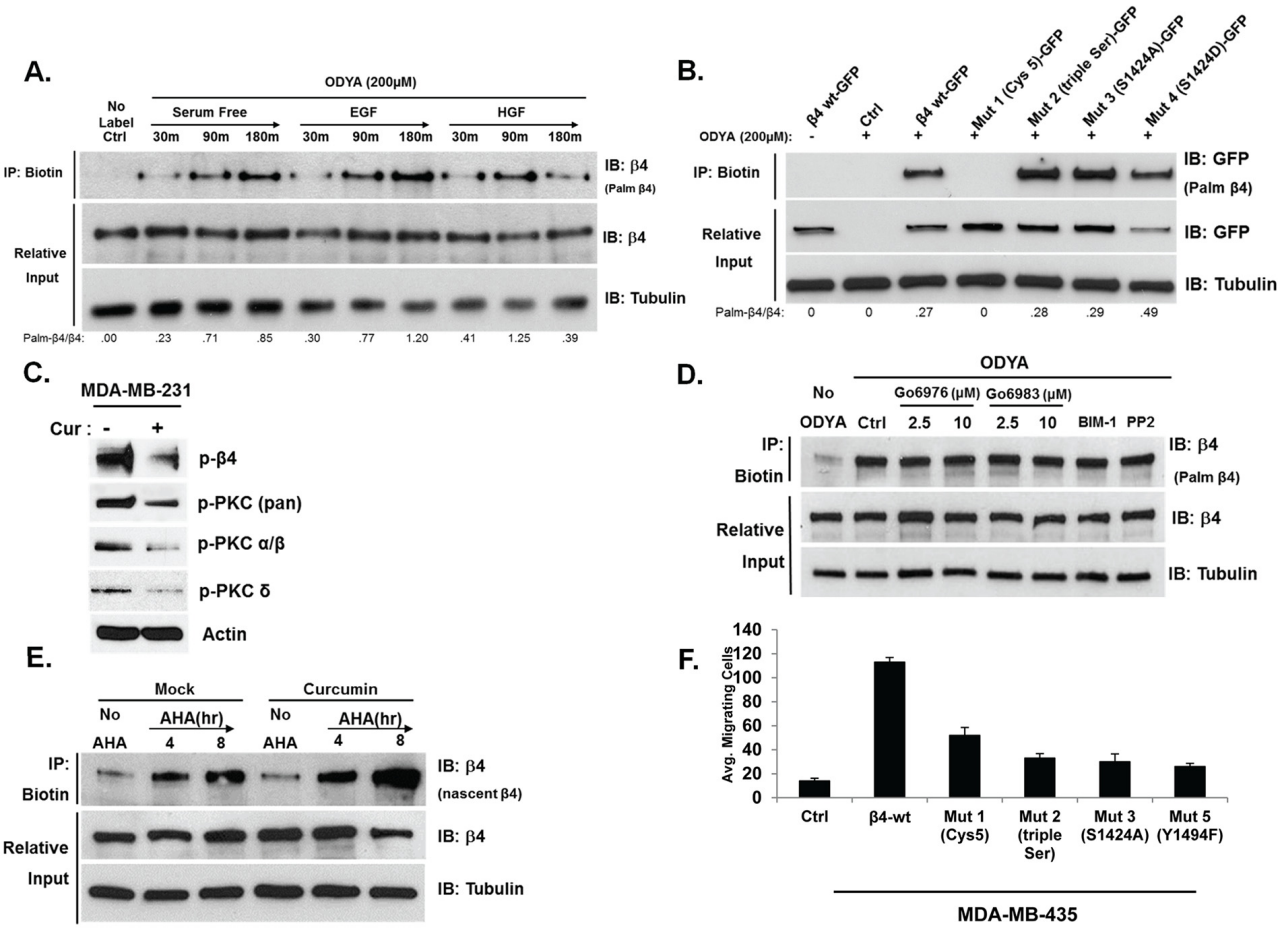
Integrin β4 palmitoylation does not require growth factor stimulation or phosphorylation of its key Ser or Tyr residues. (A)MDA-MB-231 cells were incubated in the presence of absence of 17-ODYA for the indicated times with or without 30 ng/ml HGF or 100 ng/ml EGF. (B) MDA-MB-231 cells were transiently transfected with Wild-type β4-GFP, five Cysteine mutant (Mut 1: C/A 732,736,738,739,742), triple Serine mutant (Mut 2: S/A 1356,1382,1364) or single Serine mutants (Mut 3: S/A 1424 and Mut 4: S/D 1424) prior to labeling with 17-ODYA. (C) MDA-MB-231 cells incubated with 10 μM curcumin for 24 hours. Lysates were taken and western blot analysis performed immunoblotting for the indicated proteins. (D) MDA-MB-231 cells were treated with Go6976 (2.5, 10 μM), Go6983 (2.5, 10 μM), BIM-1 (2.5 μM), or PP2 (10 μM) 1 hour prior to during 17-ODYA labeling for 5 hours. (E) MDA-MB-231 cells were incubated with or without azido-homoalanine for the given time points in the presence or absence of 15 μM curcumin to label newly-synthesized protein. (A,B,D,E) Labeled proteins were reacted with biotin-azide via click chemistry, isolated by streptavidin-sepharose, and analyzed by western blotting with indicated antibody to assess palmitoylation. Representative blots of 3 independent experiments are displayed with relative input protein included. (F) MDA-MB-435 cells were transfected with the indicated myc- tagged integrin β4 construct. The ability of the mutants to migrated toward 100 nM LPA was measuring using a transwell assay. Migration was quantified by counting cells that migrated to the lower surface of the membrane using bright-field optics. Columns, mean from three independent experiments.

Given previous reports that curcumin can block several phosphorylation signaling cascades, it was possible curcumin blocked palmitoylation indirectly by interfering with upstream signaling pathways [23]. To define the mechanism by which curcumin blocks palmitoylation, we investigated the kinetics of ITG β4 palmitoylation in relation to growth factor stimulation and phosphorylation status. Previous reports have demonstrated that ITG β4 activity and trafficking is affected by epidermal growth factor (EGF) and hepatocyte growth factor (HGF) stimulation through protein kinase C (PKC) and Src-family kinases [6–8, 10, 11, 18]. To assess whether the kinetics of ITG β4 palmitoylation are even altered in response to these signaling events, MDA-MB-231 cells were incubated with or without 17-ODYA in the presence or absence of EGF or HGF over the indicated time points. As shown in Fig 4A, the level of ITG β4 palmitoylation increased steadily even in the absence of growth factor stimulation. No fluctuations in the rate of 17-ODYA incorporation were observed over time with or without EGF or HGF stimulation (Fig 4A). A slight, yet inconsistent, reduction of ITG β4 palmitoylation was observed at later time points. To further assess the involvement of ITG β4 phosphorylation on the dynamics of its palmitoylation, we transfected GFP-tagged WT ITG β4; or phosphorylation mutants of ITG β4, including serine mutants (triple Ser: S/A 1356,1382,1364; or S/A, S/D 1424); or a palmitoylation defective five cysteine mutant of ITG β4 (Cys 5: C/A 732,736,738,739,742) into MDA-MB-231 and incubated with 17-ODYA. As a negative control, the cys 5 mutant was not labeled by 17-ODYA, confirming the authenticity of the assay and that these 5 cysteines are the palmitoylation sites within ITG β4 (Fig 4B). In contrast, neither loss nor gain of function mutations of key ITG β4 serine residues affected ITG β4 palmitoylation (Fig 4B).

Knowing that PKC and Src-family kinases are inhibited by curcumin (Fig 4C) and that these pathways are upstream of ITG β4 mobilization, we tested more closely the affect of their inhibition on ITG β4 palmitoylation. MDA-MB-231 cells were pretreated with pharmacological inhibitors of PKC isoforms Src-family kinases including Go6976 (an inhibitor of the conventional isoforms of PKC-α, -β, -γ), Go6983 (an inhibitor of PKC-α, -β, -δ, -γ isoforms), BIM-1 (an inhibitor of PKC pan), and PP2 (a Src-family kinase inhibitor) in the presence of 17-ODYA. As indicated in Fig 4D, neither PKC nor Src inhibition affected ITG β4 palmitoylation. Taken together these results suggest that ITG β4 palmitoylation is not influenced by growth factor stimulation, phosphorylation status or upstream signaling in general.

Given that ITG β4 palmitoylation seems to occur upon synthesis and this attachment remains stable despite activating events, we tested whether curcumin was affecting the rate of β4 synthesis which could account for the observed reduced palmitoylation. Click chemistry based assessment of ITG β4 synthesis in MDA-MB-231 cells revealed no consequential change to the rate of its synthesis in the presence of curcumin (Fig 4E).

To demonstrate the effect each mutant construct has on cell motility, ITG β4-null MDA-MB-435 breast cancer cells were transfected and assayed for ability to migrate through collagen-coated transwell inserts. As shown in Fig 4F, exogenous expression of wt ITG β4 provides a significant increase in migratory potential. However, mutations preventing the palmitoylation of ITG β4 (Cys 5) greatly reduce this enhancement similar to phosphorylation mutants that inhibit ITG β4 signaling capacity (triple Ser, S1424A, and Y1494F). Taken together, these data indicate that phosphorylation and palmitoylation of ITG β4 are important for its affect on cell motility; however, palmitoylation of ITG β4 is not dependent on its phosphorylation.

### Curcumin inhibits the palmitoylation of a subset of proteins without indiscriminate blocking of global cysteine acylation

**Fig 5:**
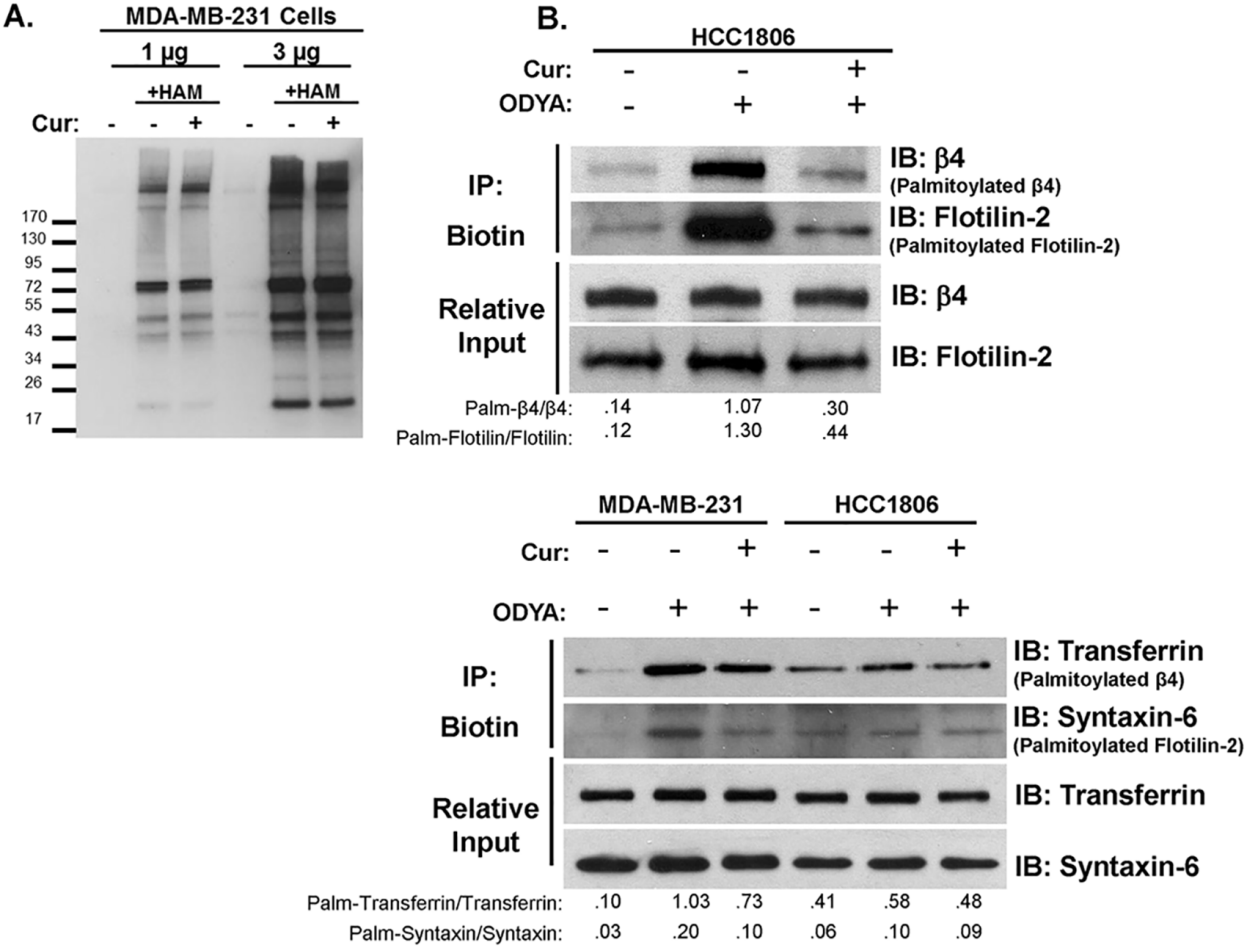
Curcumin does not indiscriminately block global cysteine modifications, but does reduce palmitoylation of proteins beside Integrin β4. (A) Acyl-biotin exchange was performed on protein from MDA-MB-231 cells treated with or without 15 μM curcumin for 18 hours. The presence or absence of hydroxylamine (HAM) during the reaction is used as a control for reaction specificity. Western blot analysis with streptavidin-HRP is shown depicting the banding pattern of S-acylated proteins from 1 or 3 mg of total protein. (B) HCC1806 or MDA-MB-231 cells (upper and lower panels where indicated) were pretreated with 15 μM curcumin for 1 hr prior to and during 5hr 17-ODYA labeling. Labeled proteins were reacted to biotin-azide via click chemistry and biotinylated proteins were isolated using streptavidin-sepharose. Palmitoylated protein was detected by western blot analysis using indicated antibodies. Representative blots of 3 independent experiments are displayed with relative input protein included and densitometry in arbitrary units.

In order to determine how selective curcumin was at inhibiting protein palmitoylation, we performed an acyl-biotin exchange technique to observe changes to global protein cysteine modifications in MDA-MB-231 cells following 24 hour incubation in the presence or absence of curcumin. In this assay, acyl-groups thioester-linked to cysteine residues are exchanged for biotin. Blotting for biotinylated protein by western blot revealed a banding pattern of whole cell acylated proteins that changes little when taken from MDA-MB-231 cells treatment with curcumin (Fig 5A). Exclusion of the hydroxylamine (HAM) treatment step from the acyl-biotin reaction is included as a negative control to indicate specificity of the biotin conjugation for only acylated cysteine residues (HAM sensitive). Two protein loads are included for clarity of the banding pattern. Somewhat surprisingly, these results further support that curcumin does not indiscriminately inhibit all, or even a large portion of, protein palmitoylation. However, immunoblotting for flotilin-2, a well characterized protein palmitoylated by DHHC5, as well as transferrin receptor or syntaxin-6, palmitoylated by an undetermined DHHC enzyme, in samples from 17-ODYA-labeled MDA-MB-231 (flotilin-2 not detected) or HCC1806 cells (Fig 5B upper and lower panels), indicated that curcumin was also able to reduce palmitoylation of additional proteins and thus is not exclusively selective for ITG β4 [16, 24–26]. Under these conditions and labeling time period, in HCC1806 cells, syntaxin-6 was not detected palmitoylated and transferrin receptor was only modestly palmitoylated, as indicated by minimal enrichment over No ODYA background.

### Curcumin reduces autoacylation of the ITG β4 palmitoylating enzyme DHHC3

**Fig 6:**
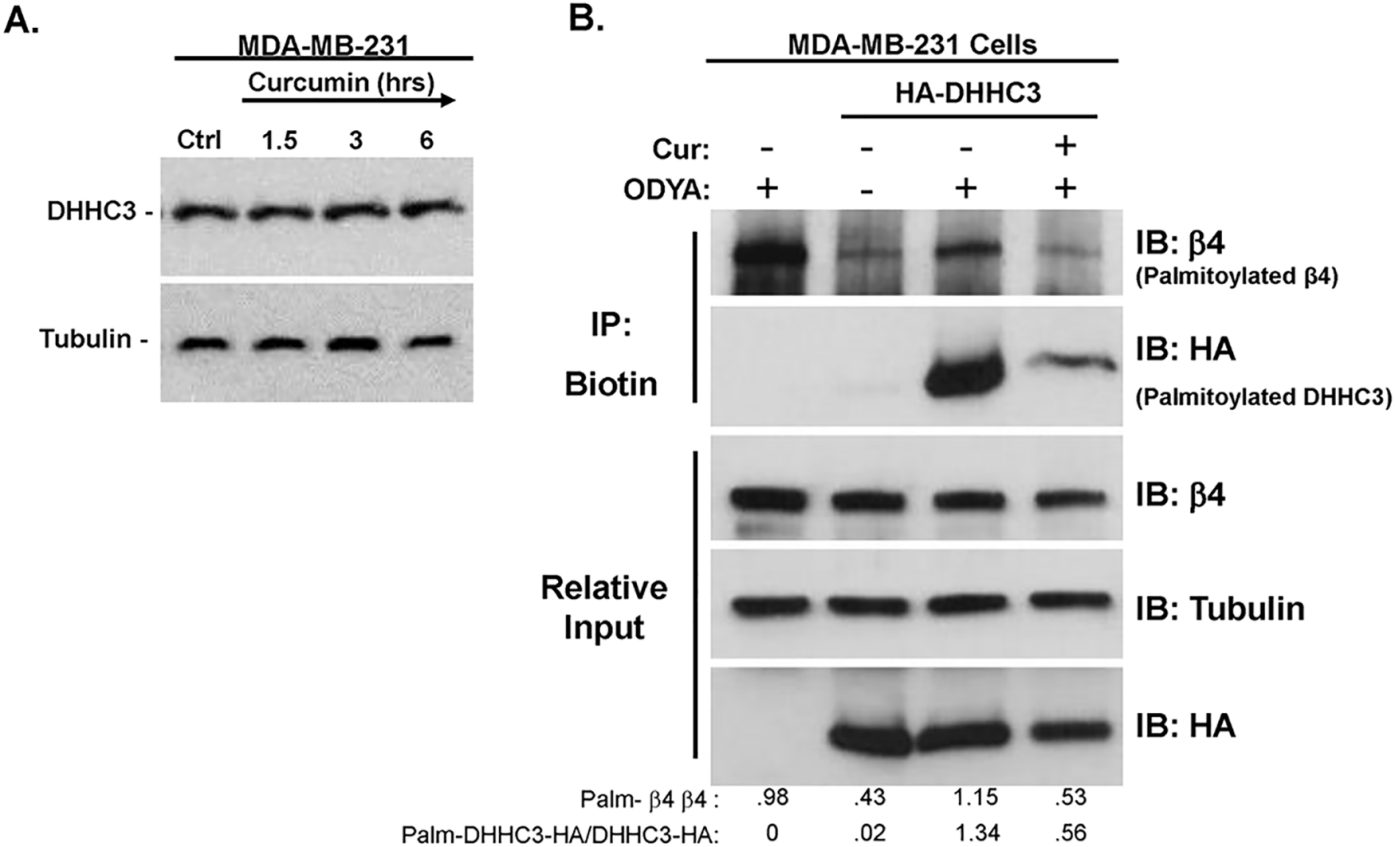
Curcumin can block the autoacylation intermediate step of DHHC3 palmitoylating enzyme activity. (A) MDA-MB-231 cells were incubated with 15 μM curcumin over the indicated time points. Lysates were taken and western blot analysis was performed using indicated antibodies. (B) MDA-MB-231 cells transiently transfected with HA-tagged DHHC3 were pretreated with 15 μM curcumin for 1 hr prior to and during 5hr 17-ODYA labeling. Labeled proteins were reacted to biotin-azide via click chemistry and biotinylated proteins were isolated using streptavidin-sepharose. Palmitoylated protein was detected by western blot analysis using indicated antibodies. Representative blots of 3 independent experiments are displayed with relative input protein included and densitometry in arbitrary units.

To begin to define the mechanism by which curcumin interferes with palmitoylation of ITG β4, and likely a subset of other proteins, we tested the possibility that curcumin inhibits the activity of acyltransferases. The acyltransferase DHHC3 is required for palmitoylation of ITG β4 [13]. It is one of 22 members of the palmitoyl acytransferase (PAT) family of proteins which transfer palmitate as palmitoyl-CoA to cysteines of substrate proteins [13, 27, 28]. This reaction requires an autoacylation intermediate step prior to transfer of the acyl-group to the substrate protein. We tested the effect of curcumin on DHHC3 expression and activity (autoacylation as an indicator). Western blot analysis of MDA-MB-231 cells treated with curcumin over the indicated time points revealed no change in the levels of DHHC3 protein (Fig 6A). However, when MDA-MB-231 cells transfected with HA-tagged DHHC3 (to overcome limiting expression levels of the native form) were incubated with 17-ODYA for 5 hours in the presence of curcumin we found a great reduction in the palmitoylation of both native (data not shown) and HA-tagged DHHC3 (Fig 6B). These data suggest that curcumin is able to block the intermediate autoacylation step of DHHC3 activity which could account for the effects of curcumin on select proteins including ITG β4.

## Discussion

ITG β4 is a transmembrane receptor for extracellular matrix components that has been linked with multiple cancer-associated phenotypes. Previous reports have demonstrated that palmitoylation of a series of cysteine residues influences function of this receptor either through membrane domain localization or protein complex formation [8,14]. Given the importance of this receptor in cancer cell growth, motility, and invasion; much effort has gone into finding ways to inhibit its function [1,2,3,20]. Our previous reports have demonstrated the ability of the polyphenol curcumin to disrupt trafficking of ITG β4 into lipid rafts and prevent its initiation of a motile phenotype. We sought to better understand the mechanism by which curcumin affected ITG β4 function, and in so doing, discovered a potential unifying mechanism for the seemingly pleiotropic effects of curcumin described in the literature.

Published reports have provided evidence that the unsaturated α,β-carbonyl groups of curcumin facilitate binding to the nucleophilic sulfhydryl group of cysteine residues [22]. Cysteine residues being the site of S-palmitoylation, provides rationale for curcumin acting as an inhibitor of this post-translational modification. This hypothesis was tested using click chemistry-based biotin labeling of palmitoylated proteins followed by a whole cell lysate biotin pull-down. For all cell lines tested, curcumin was able to block, but not reverse, the palmitoylation of ITG β4. While testing this hypothesis, we also observed that a cell line’s propensity for invasion seemed to trend with the extent to which ITG β4 was palmitoylated in that cell line under basal growth conditions. This potentially impactful observation will be elaborated in future studies. In agreement with the original rationale, we found that a structural analog of curcumin with a saturated aliphatic chain was a much weaker inhibitor of ITG β4 palmitoylation indicating the importance of the unsaturated α,β-carbonyl groups.

Little is known about the dynamics of ITG β4 palmitoylation, and to better understand how curcumin could act to block this event, it was first necessary to test possible upstream influences. These experiments provided important negative results demonstrating the likelihood that ITG β4 is stably palmitoylated along the biosynthetic pathway and is influenced little by signaling events. Pharmacological inhibition of upstream signaling pathways, including PKC and Src-family kinases, had no effect on ITG β4 palmitoylation. Similarly, loss and gain-of-function mutations of key ITG β4 phosphorylation sites had no effect on its palmitoylation. Growth factor stimulation did not substantially change the kinetics of ITG β4 palmitoylation; however, a marginal reduction was consistently observed at longer time points following HGF treatment. These data indicate that curcumin does not block ITG β4 palmitoylation by inhibition of upstream phosphorylation cascades. The importance of these findings is highlighted by the data demonstrating ITG β4 palmitoylation and phosphorylation are necessary for this integrin to promote cell motility; however its phosphorylation is not a prerequisite for its palmitoylation.

S-palmitoylation is catalyzed by the DHHC-family of palmitoyl acyltransferases with substrate specificity differing between the 22 distinct DHHC-motif containing proteins. The catalytic activity of DHHC proteins requires autoacylation of the cysteine within the DHHC motif prior to transfer of the palmitate to substrate proteins. Analyzing the acylation of DHHC3, we found that curcumin was able to greatly reduce this intermediate step, and thereby prevent the palmitoylation of ITG β4. In overexpressing DHHC3 one might expect elevated levels of palmitoylated ITG β4; however, given our observed seemingly co-translational palmitoylation of ITG β4 we wouldn’t expect this to be true.

We performed an acyl-biotin exchange technique to globally assess thioester-linked cysteine modifications and ask how promiscuously curcumin affected protein palmitoylation. The banding pattern of whole cell palmitoylated proteins between treated and untreated cells was unappreciable indicating a limited portion of proteins were affected and supporting a substantial level of specificity for the effect of curcumin. However, we found that palmitoylation of flotilin-2, syntaxin-6, and the transferrin receptor were also blocked by curcumin treatment. These results suggest that curcumin is unlikely to indiscriminately block all protein lipidation, but demonstrates some level of specificity for select proteins through inhibition of at least two DHHC enzymes. We hypothesize the nucleophilic cysteine of the DHHC motif of some, if not all, PATs is susceptible to covalent bond formation with the unsaturated α,β-carbonyl groups of curcumin, thereby blocking enzymatic palmitoylation of select proteins. It may be possible that curcumin binds to more than just DHHC proteins to block palmitoylation; however, one would expect that such a level of indiscriminate blockage, particularly at cysteine residues, would have been detected by the acyl-biotin exchange experiment. Future studies will more directly test this hypothesis to determine the extent of curcumin specificity for PATs.

In conclusion, we have provided evidence that the palmitoylation status of ITG β4 is important for cancer cell invasion and that palmitoylation of ITG β4 is a constitutive and stable event that does not seem to be influenced by upstream signaling events. These data support ITG β4 palmitoylation being necessary for proper structure and function, but having little dynamic regulatory responsibility with regard to activation and subcellular trafficking. Moreover, identifying curcumin as an inhibitor of S-palmitoylation with some selectivity establishes a possible unifying mechanism for at least some of curcumin’s seemingly pleiotropic effects.
